# Meeting Report: Summary of *IARC Monographs* on Formaldehyde, 2-Butoxyethanol, and 1-*tert*-Butoxy-2-Propanol

**DOI:** 10.1289/ehp.7542

**Published:** 2005-05-12

**Authors:** Vincent James Cogliano, Yann Grosse, Robert A. Baan, Kurt Straif, Marie Béatrice Secretan, Fatiha El Ghissassi, the Working Group for Volume 88

**Affiliations:** International Agency for Research on Cancer, Lyon, France

**Keywords:** 1-*tert*-butoxy-2-propanol, 2-butoxyethanol, carcinogen, formaldehyde, glycol ethers, hazard identification, *IARC Monographs*, leukemia, nasopharyngeal cancer, sinonasal cancer

## Abstract

An international, interdisciplinary working group of expert scientists met in June 2004 to develop *IARC Monographs on the Evaluation of the Carcinogenic Risk of Chemicals to Humans* (*IARC Monographs*) on formaldehyde, 2-butoxyethanol, and 1-*tert*-butoxy-2-propanol. Each *IARC Monograph* includes a critical review of the pertinent scientific literature and an evaluation of an agent’s potential to cause cancer in humans. After a thorough discussion of the epidemiologic, experimental, and other relevant data, the working group concluded that formaldehyde is carcinogenic to humans, based on sufficient evidence in humans and in experimental animals. In the epidemiologic studies, there was sufficient evidence that formaldehyde causes nasopharyngeal cancer, “strong but not sufficient” evidence of leukemia, and limited evidence of sinonasal cancer. The working group also concluded that 2-butoxyethanol and 1-*tert*-butoxy-2-propanol are not classifiable as to their carcinogenicity to humans, each having limited evidence in experimental animals and inadequate evidence in humans. These three evaluations and the supporting data will be-published as Volume 88 of the *IARC Monographs*.

Twenty-six scientists from 10 countries met at the International Agency for Research on Cancer (IARC) in June 2004 to develop *IARC Monographs on the Evaluation of the Carcinogenic Risk of Chemicals to Humans* (*IARC Monographs*) on formaldehyde, 2-butoxyethanol, and 1-*tert*-butoxy-2-propanol (IARC, in press). This is the fourth IARC evaluation of formaldehyde and the first of the glycol ethers.

Formaldehyde is widely used in resins that bind wood products, pulp and paper, and glasswool and rockwool insulation. It is also used in plastics and coatings, textile finishing, and chemical manufacturing and as a disinfectant and preservative. High concentrations can be found in some work environments, and much lower concentrations in homes.

2-Butoxyethanol is a glycol ether widely used as a solvent in paints, paint thinners, glass-cleaning and surface-cleaning products (especially in the printing and silk-screening industries), and personal-care and other personal products and as a chemical intermediate. General-population exposure can occur through the use of consumer products, particularly cleaning agents.

1-*tert*-Butoxy-2-propanol is a glycol ether that has found increasing use as a solvent in coatings, glass-cleaning and surface-cleaning products, inks, adhesives, and nail-polish lacquers.

## Materials and Methods

IARC convenes an international, interdisciplinary working group of expert scientists to develop each volume of the *IARC Monographs*. The working group writes a critical review of the pertinent scientific literature (published articles, articles accepted for publication, and publicly available documents from government agencies) and a consensus evaluation of each agent’s potential to cause cancer in humans.

The *IARC Monographs* are developed during an 8-day meeting whose objectives are review and consensus. Before the meeting, each member of the working group writes a portion of the critical review. At the meeting, four subgroups (exposure, cancer in humans, cancer in experimental animals, and mechanistic and other relevant data) review these drafts and develop consensus subgroup drafts. Then the working group meets in plenary session to review the subgroup drafts and develop a consensus evaluation. After the meeting, IARC scientists review the final draft for accuracy and clarity before publication.

The evaluation is developed in steps (IARC 2005). The subgroup of epidemiologists proposes an evaluation of the evidence of cancer in humans as sufficient evidence, limited evidence, inadequate evidence, or evidence suggesting lack of carcinogenicity. A subgroup of toxicologists and pathologists proposes an evaluation of the evidence of cancer in experimental animals, choosing one of the same descriptors. Combination of these two partial evaluations yields a preliminary default evaluation that the agent is one of the following: group 1, carcinogenic to humans; group 2A, probably carcinogenic to humans; group 2B, possibly carcinogenic to humans; group 3, not classifiable as to its carcinogenicity to humans; or group 4, probably not carcinogenic to humans.

When the epidemiologic evidence is sufficient, the final evaluation is carcinogenic to humans, regardless of the experimental evidence. In other cases, the mechanistic and other relevant data are considered to determine whether the default evaluation should be modified upward or downward. A subgroup of experts in cancer mechanisms assesses the strength of the mechanistic data and whether the mechanisms of tumor formation in experimental animals can operate in humans. The overall evaluation is a matter of scientific judgment, reflecting the combined weight of the evidence.

Working groups are selected to invite the best-qualified experts and to avoid real or apparent conflicts of interests. Consideration is given also to demographic diversity and a balanced representation of all scientific views. Each potential participant submits a Declaration of Interests [World Health Organization (WHO) 2005], which IARC assesses to determine whether there is a conflict that warrants some limitation on participation. An expert with a real or apparent conflict of interest may not serve as chairperson, draft text discussing cancer data, or participate in the evaluations. IARC strives to ensure that the working group is free from all attempts at interference, before and during the meeting. This includes lobbying, written materials, and meals or other favors offered by interested parties. Working group members are asked not to discuss the subject matter with anyone outside the meeting and to report all attempts at interference (Cogliano et al. 2004).

## Results

### Formaldehyde.

There was a statistically significant excess of deaths from nasopharyngeal cancer in the largest and most informative cohort study of industrial workers (Hauptmann et al. 2004), with statistically significant exposure–response relationships for peak and cumulative exposure. An excess of deaths from nasopharyngeal cancer was also observed in a proportionate mortality analysis of the largest U.S. cohort of embalmers (Hayes et al. 1990), and an excess of cases of nasopharyngeal cancer was observed in a Danish study of proportionate cancer incidence among workers at companies that manufactured or used formaldehyde (Hansen and Olsen 1995). Although other cohort studies reported fewer cases of nasopharyngeal cancer than expected (Coggon et al. 2003; Pinkerton et al. 2004; Walrath and Fraumeni 1983), the working group noted that the deficits were small and the studies had low power to detect an effect on nasopharyngeal cancer. Of seven case–control studies of nasopharyngeal cancer (Armstrong et al. 2000; Hildesheim et al. 2001; Olsen et al. 1984; Roush et al. 1987; Vaughan et al. 1986, 2000; West et al. 1993), five found elevations of risk for exposure to formaldehyde. The working group considered it “improbable that all of the positive findings for nasopharyngeal cancer that were reported from the epidemiologic studies, and particularly from the large study of industrial workers in the United States, could be explained by bias or unrecognized confounding effects.” The working group concluded that these studies provide “sufficient epidemiological evidence that formaldehyde causes nasopharyngeal cancer in humans.”

Excess mortality from leukemia, primarily of the myeloid type, has been observed relatively consistently in six of seven studies of embalmers, funeral parlor workers, pathologists, and anatomists (Hall et al. 1991; Hayes et al. 1990; Levine et al. 1984; Logue et al. 1986; Stroup et al. 1986; Walrath and Fraumeni 1983, 1984). A recent meta-analysis found that, overall, the relative risk for leukemia in these workers was increased and did not vary significantly among studies (Collins and Lineker 2004). There had been speculation that these findings might be explained by viruses; however, the working group found little evidence that these occupations have a higher incidence of viral infections or that viruses have a causal role in myeloid leukemia. Until recently, these leukemia findings received little attention because excess leukemia had not been observed in the studies of industrial workers. There is now, however, some evidence for an association between formaldehyde exposure and leukemia in the recent updates of two of the three major industrial cohorts. A statistically significant exposure–response relationship was observed for leukemia and, particularly, for myeloid leukemia in the study of industrial workers in the United States, based on peak exposure and, to a lesser degree, on average intensity of exposure to formaldehyde (Hauptmann et al. 2003).

There was no excess mortality from leukemia when the industrial workers were compared with the general U.S. population, but a comparison with the general population may be biased. In another study, excess mortality from leukemia was found in the recent update of garment workers in the United States (Pinkerton et al. 2004). This excess was statistically significant among workers with a longer duration of exposure and follow-up. In contrast, the updated study of industrial workers in the United Kingdom did not find excess mortality from leukemia (Coggon et al. 2003). This high-quality study had sufficient size and follow-up to have reasonable power for detecting an excess of leukemia, but it did not report on peak exposures or the risk of myeloid leukemia specifically. The working group concluded, “In summary, there is strong but not sufficient evidence for a causal association between leukaemia and occupational exposure to formaldehyde.” This conclusion, falling between sufficient and limited evidence, was based on a consistently increased risk in studies of embalmers, funeral parlor workers, pathologists, and anatomists and was present in two of the three most informative studies of industrial workers.

Several case–control studies have investigated the relationship between formaldehyde exposure and sinonasal cancer. A pooled analysis of 12 studies showed an increased risk of adenocarcinoma in men and women thought never to have been exposed to wood dust or leather dust, with an exposure–response trend for an index of cumulative exposure (Luce et al. 2002). One other case–control study (Olsen and Asnaes 1986) and a proportionate incidence study (Hansen and Olsen 1995) showed an increased risk of sinonasal cancer, particularly squamous cell carcinoma. Against these largely positive findings, the three most informative cohort studies of industrial workers showed no excesses of sinonasal cancer (Coggon et al. 2003; Hauptmann et al. 2004; Pinkerton et al. 2004). The working group noted that most studies did not distinguish tumors as originating in the nose or sinuses; thus, an increased risk of nasal cancer would be diluted if there were no corresponding effect on the sinuses. In the case–control studies, the working group also noted the potential for confounding by wood dust exposure, which is associated with adenocarcinoma. The working group concluded that there is limited evidence that formaldehyde causes sinonasal cancer in humans.

In experimental animals, several studies have shown that inhalation exposure induces squamous cell carcinomas of the nasal cavities in rats (Albert et al. 1982; Feron et al. 1988; Gibson 1984; Kamata et al. 1997; Kerns et al. 1983; Monticello et al. 1996; Morgan et al. 1986; Sellakumar et al. 1985; Woutersen et al. 1989), although single studies in mice (Kerns et al. 1983) and hamsters (Dalbey 1982) showed no carcinogenic effects. Four studies of formaldehyde administered to rats in drinking water gave varying results: One showed an increased incidence of forestomach papillomas in male rats (Takahashi et al. 1986); a second showed an increased incidence of gastrointestinal leiomyosarcomas in female rats and in both sexes combined (Soffritti et al. 1989); a third showed increased incidences of total malignant tumors, lymphomas and leukemias, and testicular interstitial-cell adenomas in male rats (Soffritti et al. 2002); whereas a fourth did not show a carcinogenic effect (Til et al. 1989). Formaldehyde also showed co-carcinogenic effects by inhalation, ingestion, and dermal exposure (Dalbey 1982; Iverson 1986; Takahashi et al. 1986).

The toxicokinetics of inhaled formaldehyde have been well studied (Agency for Toxic Substances and Disease Registry 1999). More than 90% of inhaled formaldehyde is absorbed in the upper respiratory tract (Heck et al. 1985). Absorbed formaldehyde can be oxidized to formate and carbon dioxide or can be incorporated into biologic macromolecules. Formaldehyde has a half-life of about 1 min in rat plasma (Rietbrock 1965). Inhalation exposure has not been found to alter the endogenous concentration of formaldehyde in the blood of rats, monkeys, or humans (Casanova et al. 1988; Heck et al. 1983, 1985). Oral exposure to ^14^C-formaldehyde resulted in some excretion in urine and feces within 12 hr (Galli et al. 1983). Dermal application of ^14^C-formaldehyde resulted in some urinary excretion in rats and monkeys (Jeffcoat et al. 1983).

Evidence shows that formaldehyde is genotoxic in multiple *in vitro* models and in exposed humans and laboratory animals. Human studies reported increased DNA–protein crosslinks in workers exposed to formaldehyde (Shaham et al. 1996, 2003), and this is consistent with studies in laboratory rats and monkeys. Cellular proliferation increases considerably at concentrations > 6 ppm and amplifies the genotoxic effects of formaldehyde. The working group concluded, “The current data indicate that both genotoxicity and cytotoxicity play important roles in the carcinogenesis of formaldehyde in nasal tissues.” On the other hand, with respect to the potential for formaldehyde to induce leukemia, the working group was not aware of any good rodent models for acute myeloid leukemia in humans. Several possible mechanisms were considered, such as clastogenic damage to circulating stem cells. There is a single study reporting cytogenetic abnormalities in the bone marrow of rats inhaling formaldehyde (Kitaeva et al. 1990). The working group concluded, “Based on the data available at this time, it was not possible to identify a mechanism for the induction of myeloid leukaemia in humans.” This is an area needing more research.

The working group concluded that formaldehyde is carcinogenic to humans (group 1), based on sufficient evidence in humans and sufficient evidence in experimental animals. Based on the information now available, this classification is higher than those of previous IARC evaluations (IARC 1982, 1987, 1995).

### 2-Butoxyethanol.

2-Butoxyethanol was tested for carcinogenicity by inhalation exposure in male and female mice and rats [National Toxicology Program (NTP) 2000]. Clear increases in tumor incidence were observed only in mice. In male mice exposed to 2-butoxyethanol, there was a dose-related increase in the incidence of hemangiosarcomas of the liver. In female mice, there was a dose-related increase in the incidences of combined forestomach squamous-cell papillomas and carcinomas (mainly papillomas). In female rats, there was a positive trend in the occurrence of benign or malignant pheochromocytomas (mainly benign) of the adrenal medulla, but this equivocal result could not be attributed with confidence to exposure to 2-butoxyethanol. No increases were observed in male rats. The epidemiologic data were inadequate for this compound.

Regarding mechanisms of carcinogenesis, the working group considered that hemolysis and associated oxidative stress in the liver have been proposed to be linked to the induction of mouse liver neoplasia. They also considered that, in view of lower sensitivity to hemolysis of human erythrocytes and higher human liver concentrations of the antioxidant vitamin E, the induction of liver tumors in humans would be improbable through this pathway, but it was noted that other potential mechanisms have not been investigated. The working group observed that the mouse forestomach tumors are associated with high local exposure to 2-butoxyethanol and high local concentrations of the toxic metabolite 2-butoxyacetic acid.

The working group concluded that 2-butoxyethanol is not classifiable as to its carcinogenicity to humans (group 3), with limited evidence in experimental animals and inadequate evidence in humans.

### 1-tert-Butoxy-2-propanol.

1-*tert*-Butoxy-2-propanol was tested for carcinogenicity by inhalation exposure in male and female mice and rats (Doi et al. 2004; NTP 2003). In a single study in both male and female mice, a dose-related increase in the combined incidence of liver tumors (hepatocellular adenomas and carcinomas), including hepatoblastomas, was observed. When hepatocellular carcinomas and hepatoblastomas were combined, there was a significant trend for the increase in malignant tumors in females. In male rats, there were marginal, nonsignificant increases in the incidences of renal tubule adenomas (with one carcinoma at the highest dose) and hepatocellular adenomas, but these findings were considered to be equivocal. In female rats, there were no dose-related increases in tumor incidence. No epidemiologic data were available for this compound.

With regard to mechanisms of carcinogenesis, the working group found the available data inadequate to elucidate a potential mechanism for the mouse liver tumors. They found the renal effects largely consistent with the α_2u_-globulin–associated nephropathy that occurs in male rats, but concluded that the available evidence satisfies only some, but not all, of the IARC criteria for the mechanism associated with accumulation of α_2u_-globulin. Regarding the potential for genotoxic effects, the working group was not able to draw any meaningful conclusion in view of the scarcity of the data available.

The working group concluded that 1-*tert*-butoxy-2-propanol is not classifiable as to its carcinogenicity to humans (group 3), with limited evidence in experimental animals and inadequate evidence in humans.

## Discussion

A theme common to these three evaluations is the consideration of mechanistic information to develop and evaluate hypotheses about the sequence of steps leading to the induction of tumors in experimental animals. The hypothesized mechanisms described in these evaluations provide an interesting set of cases that range from a vast literature on respiratory-tract tumors in rats induced by inhalation of formaldehyde to some more tentative hypotheses about the various tumors observed in animals after exposure to glycol ethers. Both types of mechanistic data sets were of use in the evaluation process.

The evaluation of formaldehyde as carcinogenic to humans shows the importance of mechanistic information in the classification of carcinogens. For the nasopharyngeal tumors, the working group discussed the convergence of the epidemiologic, experimental, and mechanistic evidence. If the evidence in humans had been less than sufficient, the strong mechanistic evidence in exposed humans and sufficient evidence in experimental animals might still have led to classification as group 1. The extensive mechanistic data for formaldehyde-induced respiratory cancer provide strong support for the empirical observation of nasopharyngeal cancer in humans, although computer models that predict an anterior-to-posterior gradient of formaldehyde deposition in the upper respiratory tract would predict that formaldehyde would cause cancer in the nose as well as the nasopharynx in humans.

On the other hand, the lack of information on possible mechanisms by which formaldehyde might increase the risk of leukemia in humans tempered the interpretation of the epidemiologic data on that cancer type. The entire working group discussed at length this divergence between the epidemiologic and mechanistic conclusions for leukemia. Information to support a biologically plausible mechanism could have supported a stronger conclusion about the evidence of leukemia in humans.

In the evaluations of the glycol ethers, the working group grappled with questions of interpretation and scientific judgment. A recurring issue was the criterion for characterizing a rare tumor or an unusual set of observations that can carry greater weight than a typical bioassay result. A related matter was how to bring in additional information to resolve difficult questions—for example, how to consider the results of historical controls or alternative statistical tests. When the working group tried to, but could not, reach consensus on a question of interpretation or scientific judgment, the evaluation presented the differing positions favored by its members. For example, after thorough discussion, several members of the working group favored an evaluation of the carcinogenicity in experimental animals as sufficient for 1-*tert*-butoxy-2-propanol. This view emphasized the dose-related induction of hepatoblastoma in male and female mice, considering hepatoblastoma as a rare neoplasm with low spontaneous incidence in mice, especially in females. Most of the working group, nevertheless, considered the evidence to be limited, based on the interpretation of hepatoblastoma being a variant of hepatocellular carcinoma.

It is important to note that the evaluation of an agent as not classifiable as to its carcinogenicity to humans is not a determination of safety, with respect to both cancer and effects other than cancer. It indicates that the data did not meet the minimum standards developed by the IARC for sufficient evidence in experimental animals and suggests that further testing is needed, particularly when there is widespread human exposure or another reason for public health concern.
